# Advances in colorectal cancer screening and detection: a narrative review on biomarkers, imaging and preventive strategies

**DOI:** 10.1186/s43046-025-00277-z

**Published:** 2025-04-11

**Authors:** Adil khan, Uswa Hasana, Iman Anum Nadeem, Swara Punit Khatri, Shayan Nawaz, Qurat Ulain Makhdoom, Shahab Wazir, Kirtan Patel, Mohamd Ghaly

**Affiliations:** 1 Nowshera Medical College, Nowshera, Pakistan; 2 Fatima Jinnah Medical University, Lahore, Pakistan; 3 University College of Medicine and Dentistry Lahore, Lahore, Pakistan; 4 Gujarat Cancer Society Hospital and Research Center, Ahmedabad, India; 5 Government Medical Collegeand , New Civil Hospital, Surat, India; 6 University of Sharjah, Sharjah City, United Arab Emirates

**Keywords:** CRC screening, Colorectal cancer, Colorectal adenomas, Colorectal cancer detection, Colorectal cancer screening and detection

## Abstract

**Background:**

With the third incident rate and a second mortality rate, colorectal cancer (CRC) continues to be one of the most prevalent and deadly malignancies worldwide. Adenomas usually develop into adenocarcinomas in colorectal cancer (CRC), a process that can be halted by early detection and prevention care.

**Main body:**

Faecal immunochemical testing (FIT) and endoscopies are examples of current screening tools that dramatically lower the incidence and death of colorectal cancer. Current development centre on non-invasive methods that provide better accuracy and lower dangers, such as blood-based liquid biopsies and imaging modalities like CT and MR colonography. For early detection, liquid biopsies—especially those using methylated DNA tests like SEPT9—offer encouraging outcomes. Circulating tumour DNA (ctDNA) has emerged as a crucial biomarker, increasing early identification and therapy monitoring. Proteomic and metabolic indicators further improve screening by figuring out who is at high risk and keeping an eye out for recurrence. The accuracy and detection rates of polyps have increased due to advancements in imaging technologies like as artificial intelligence (AI), narrow-band imaging (NBI), and high-definition colonoscopy. The emphasis has been on preventive measures, such as chemoprevention and lifestyle modifications, dietary fibre, regular exercise, and chemoprotective drugs like aspirin have demonstrated potential in lowering the incidence of colorectal cancer. There are still issues with the global implementation of screening, including differences in access to screening between socioeconomic and racial groups. Hope for more individualized and efficient CRC screening and prevention are provided by new research on biomarkers and technological advancements like artificial intelligence and polygenic risk classification.

**Conclusion:**

With a variety of invasive and non-invasive techniques available to identify cancer early. With a variety of invasive and non-invasive techniques available to identify cancer early. To enhance prognosis and lower mortality, colorectal cancer screening has undergone tremendous advancement. Although colonoscopy and faecal immunochemical assays (FIT) are still good standards for detecting colorectal cancer (CRC), advances in liquid biopsy, proteomics, and imaging have transformed the field and offered less invasive, more precise choices, for early identification and surveillance, circulating tumour DNA (ctDNA) and other biomarkers show tremendous potential.

## Background

### Overview of colorectal cancer (CRC) screening and prevention

Colorectal cancer is one of the most common cancers worldwide. Overall CRC ranks third in terms of incidence but second in terms of mortality. The transformation from adenoma to adenocarcinoma is a slow process so a substantial proportion of CRC cases can be prevented [1]. Screening for CRC helps detect adenomas and polyps in the early stage and prevents them from transforming into CRC. It can also detect cancer in its early stage, prevent its progression, and reduce mortality [2]. Screening for colon cancer has significantly contributed to a decrease in morbidity and mortality related to colorectal cancer (CRC) in the past 2 decades [3]. There are several options used currently for colorectal cancer screening. Faecal immunochemical test (FIT) is the most used non-invasive method worldwide. FIT when performed annually or biennially yields comparable results of detecting CRC as colonoscopy once every 10 years [4]. Endoscopic screening techniques may prevent colon cancer as precancerous polyps can be removed and prevent it from transforming into CRC [5]. Flexible sigmoidoscopy helps decrease distal CRC incidence and related mortality but it has no significant effect on proximal CRC [6]. In a large prospective cohort study, it was found that with colonoscopy, with polypectomy, the incidence of CRC reduced to 43% compared to those with no lower endoscopy [7]. 

### Purpose and scope of the review

In recent years, newer techniques of blood-based CRC screening called liquid biopsy are gaining popularity. Methylated septic DNA assay is the first FDA-approved blood-based CRC screening test [8]. Electronic chromoendoscopy uses white light which highlights neoplastic lesions and is advantageous as they are accurate, fast to perform, and inexpensive [9]. MR colonography is a non-invasive screening method developed recently. It can detect metastasis and extracolonic mass without the risk of radiation exposure [4]. CT colonography is a non-invasive method used for screening. It can also report extracolonical renal or adrenal masses [4]. Another method is a colon capsule, a pill-sized camera that is swallowed which gets activated in the ileum [4]. Some programs use colonoscopy as a primary screening tool, while others use it only for diagnostic clarification after first-line less invasive tests are positive [10]. According to the latest guidelines, a colonoscopy is done every 10 years or a faecal immunochemical test (FIT) annually for normal-risk individuals, while for individuals with higher risk other specific recommendations are provided [11]. 

### Novel biomarkers for CRC Screening

#### Genetic and epigenetic biomarkers

DNA methylation alterations are frequently observed in carcinogenesis and genomic imprinting disorders, such as Angelman syndrome and Prader-Willi syndrome. These alterations are advantageous as biomarkers due to their stability and accessibility in body fluids [12, 13] making them particularly valuable for colorectal cancer screening—the third most common cancer. Although colorectal cancer is treatable if detected early, given that it often originates from precancerous polyps that can be removed before progressing to cancer, it still accounts for nearly 9% of cancer-related deaths, largely due to insufficient screening rates [14]. Currently, established DNA methylation biomarkers utilized in clinical settings for cancer screening, due to their diagnostic, prognostic, and predictive value, include BCAT1, IKZF1, SEPT9, BMP3, and NDRG4 [15]. However, only SEPT9 (in Epi proColon®) and a combination of NDRG4 and BMP3 (in Cologuard®) have received FDA approval and are used for colorectal cancer diagnosis [16, 17]. Moreover, emerging DNA methylation biomarkers associated with colorectal cancer metastasis include BDNF, FIGN, HTRA3, STAC2, TBRG4, and HCN4 [15].

Between 5 and 15% of CRCs have been found to have deficient DNA mismatch repair machinery (dMMR) and, as a consequence, high microsatellite instability (MSI) [18]. Hence, the MMR/MSI status is often employed as a biomarker in CRC patients due to its prognostic and predictive value especially in high-risk populations such as in patients with family or personal history of CRC, adenomas, and genetic syndromes [19].

#### Circulating tumour DNA (ctDNA)

Circulating tumour DNA (ctDNA) consists of DNA fragments released into the bloodstream either upon tumour cell death or through active secretion. Due to its tumour-specific genetic information, ctDNA serves as a crucial biomarker for cancer detection. Keeping in mind that a blood sample of 10 ml has to be drawn and processed within a few hours for this procedure [20]; it still offers several advantages over traditional diagnostic methods: ctDNA analysis is non-invasive, capable of detecting minimal residual disease—an area where imaging modalities like MRI and ultrasound fall short—and avoids the use of ionizing radiation, thus reducing patient risk. Various technologies can be employed to detect ctDNA in blood samples such as real-time PCR, digital PCR, next generation sequencing, and mass spectrometry [21]. However, despite these advantages, the clinical application of ctDNA necessitates further refinement. The detection process requires a substantial volume of blood and involves complex purification techniques that require enhancement. Furthermore, ctDNA analysis methods are often costly, which restricts their accessibility in clinical settings. Additionally, false positives may arise due to the insufficient sensitivity of current detection techniques, resulting in a lack of standardization [21, 22]. ctDNA’s sensitivity for detecting cancer varies with the stage and site of origin. Studies have shown that sensitivity increases with cancer stage, ranging from approximately 40% in stage I to around 80% in stage III malignancies [23]. In the case of specificity, it provides greater specificity in detecting early metastatic/recurrent disease as seen in the case of patients with surgically resected colorectal cancer where in various studies ctDNA was seen to be superior to carcinoembryonic antigen (CEA) in the detection of residual disease and early recurrence [22]. Currently, circulating tumour DNA (ctDNA) has emerged as a valuable tool for the early assessment of immune checkpoint responses and can effectively complement standard imaging studies [24]. Additionally, improved sensitivity in detecting recurrence has been observed in colorectal cancer (CRC) patients [25]. Another significant advantage of ctDNA is its capability to monitor therapeutic effects by identifying mutation-driven resistance [21].

#### Proteomic and metabolic markers: (Table 1)

**Table 1:** Important biomarkers for CRC screening and detection

Biomarker	Type	Clinical use	Sensitivity	Status	Notes
SEPT9 (Septin9, Epi proColon)	DNA methylation	Diagnostic	Moderate (67%)	FDA-approved	First FDA-approved blood test for CRC, limited early detection ability
NDRG4, BMP3	DNA methylation	Diagnostic	High (92%)	FDA-approved	Used in stool-based CRC test (e.g. Cologuard)
Circulating tumour DNA (ctDNA)	Liquid biopsy	Prognostic, therapeutic monitoring	Variable (40–80%)	Experimental	Shows promise in early recurrence detection
Carcinoembryonic antigen (CEA)	Protein marker	Prognostic	Low (~ 50%)	Widely used	Not very specific for CRC alone
CA19-9	Protein marker	Prognostic	Low	Investigational	Limited early detection ability
MicroRNAs (miRNAs) (e.g. miR-21-5p, miR-92a-3p, miR-135b-5p, miR-17–92 cluster, miR-27a-3p, miR-146a-5p)	RNA-based	Diagnostic, prognostic	High	Under research	Non-invasive biomarkers in stool and blood, promising for early CRC detection
Long non-coding RNAs (lncRNAs) (e.g. DLEU1, LINC01094, MALAT1, CASC15, CRART16, FGD5-AS1)	RNA-based	Diagnostic, therapeutic	Variable	Under research	Regulate CRC progression, metastasis, and drug resistance
Dysregulated snoRNAs (SNORA51, SNORD15B)	RNA-based	Diagnostic	High (AUC = 0.86 for CRC)	Under research	Potential for advanced CRC detection, combined with fecal hemoglobin
SCAT1, SCAT2, SCAT8	RNA-based	Diagnostic	Not specified	Under research	Upregulated in CRC tissues, potential biomarkers
Circulating tumour cells (CTCs)	Liquid biopsy	Diagnostic, prognostic	High (95.2%)	Experimental	High sensitivity for early cancer detection
Cell-free DNA (cfDNA)	Liquid biopsy	Diagnostic, prognostic	Variable	Experimental	Detects mutations and monitors recurrence
BDNF, FIGN	DNA methylation	Prognostic	Moderate	Under research	Associated with CRC metastasis
CMSmin	Risk stratification	Screening	Moderate	Under research	Reduced CRC incidence by 40% and mortality by 52% but less effective than FIT or mt-sDNA

Blood-based protein biomarkers, whether diagnostic, predictive, or prognostic, provide a non-invasive method for colorectal cancer (CRC) screening. Other biological substances such as urine and saliva can assist in diagnosis, although cancer-related biomarkers are predominantly blood-based [26].

Molecular pathways like WNT, MAPK, TGFβ, and p53; proteins like C1QP, ERGIC1, ORMDL1; and plasma biomarkers such as fibroblast growth factor 21 (FGF-21), pancreatic prohormone (PPY) along with urine markers COROC1C, RAD23B, and ARPC3 are strongly linked to the pathogenesis of CRC [27]. Proteins such as GREM1, CLSTN3, CSF2RA, and CD86 have also been strongly linked to CRC [28]. Resistant tumours have been known to show overexpression of PCBP1 protein, whereas the expression of APOE, AGT, and DBP has been linked to better survival outcomes in patients undergoing treatment with chemotherapy and bevacizumab [27]. Carcinoembryonic antigen (CEA) is the most common prognostic biomarker for CRC and helps indicate the risk of tumour recurrence. Maspin has been highlighted as an early marker for stage IV CRC [27]. Glycodeoxycholic acid (GDCA) has also showed high sensitivity and specificity as a diagnostic marker [29]. Still, CEA remains the only prognostic biomarker used in clinical practice [27]. Metabolomic analysis of fecal samples may identify biomarkers, as histamine was found at lower concentrations in inflamed mice compared to healthy ones, indicating a role in tumourigenesis [30]. 

#### Colonoscopy

High-definition (HD) colonoscopy has proven to offer only a little improvement in adenoma detection compared to standard-definition colonoscopy. Virtual CC is now integrated into HD colonoscopes through light filters like narrow-band imaging (NBI), but more research is needed on whether this advancement leads to better detection or not. The computer-aided design technologies enhance adenoma detection, but this area also needs more research before its clinical implementation [31].

#### Chromoendoscopy

The 2019 BSG and ECCO guidelines recommend high-definition (HD) endoscopy and chromoendoscopy (CE) over standard techniques. The 2019 ACG guidelines for ulcerative colitis suggest using HD with WLE (white light endoscopy) and narrow-band imaging (NBI) or CE. For invisible low-grade dysplasias, CE and biopsies within 3 months, and for high-grade, dysplasia or carcinoma, urgent repeat CE are recommended. SD CE outperforms SD WLE in dysplasia detection, but HD CE and HD WLE have similar rates [32].

#### Narrow-band imaging

Narrow-band imaging (NBI) focuses on wavelengths of 415 and 540 nm to enhance contrast for surface vessels and structures. NBI has higher adenoma detection rates compared to HD WLE and also shows improved detection for non-adenomatous polyps, sided non-adenomatous polyps, and flat polyps. However, NBI is considered less effective than chromocolonoscopy (CC) in Lynch syndrome patients, although it excels in detecting adenomas [31]. 

### Some non-invasive tests and biomarkers

Although colonoscopy is the gold standard, its high cost and invasiveness limit widespread use [33]. Blood tests like CEA, CA19-9, and Septin9 have limitations, particularly in early detection. MicroRNA (miRNA) offers promise yet is not fully ready for clinical adoption [34]. Patients prefer non-invasive tests, emphasizing the need for accessible and convenient screening methods to ensure thorough cancer detection [35]. Novel, non-invasive diagnostic methods, including DNA mutations mRNA, miRNA, gut microbes, and metabolites, as well as multiomics, are needed to improve screening [36, 37].

The multitarget stool RNA test is a non-invasive screening tool for colorectal cancer (CRC) and advanced adenomas. It combines a faecal immunochemical test (FIT), eight RNA transcript concentrations, and participant-reported smoking status [38, 39]**.**

Multitarget stool RNA (mt-sRNA) screening strategy every 3 years outperformed multitarget stool DNA 2.0 (mt-sDNA 2.0) and cell-free DNA (cf-DNA) while remaining a cost-effective option [40]**.**

Barnell et al. assessed the unapproved mt-sRNA test in 8920 patients, showing it detected 94.4% of CRC cases and 45.9% of advanced adenomas. It outperformed FIT in CRC sensitivity but had lower specificity. Hence, mt-sRNA demonstrated higher sensitivity than FIT for CRC and advanced adenomas but had lower specificity for negative colonoscopy results [41]**.**

MicroRNAs (miRNAs) like miR-21-5p, miR-92a-3p, and miR-135b-5p are also promising noninvasive biomarkers for CRC detection. miR-21-5p and miR-92a-3p demonstrate high sensitivity and specificity in stool and blood samples, while miR-135b-5p excels in stool sensitivity. The miR-17–92 cluster and other miRNAs like miR-27a-3p and miR-146a-5p also aid CRC detection. Combining these miRNAs can enhance CRC screening, especially for precancerous lesions [42]**.**

RNA sequencing identified potential cfRNA candidates in phase 1, which were then validated using targeted capture sequencing and RT-qPCR in phases 2 and 3. Although 895 exons were differentially expressed in cancers, fewer markers were successfully validated than anticipated [43]**.**

Long non-coding RNAs (lncRNAs) like DLEU1, LINC01094, MALAT1, CASC15, CRART16, and FGD5-AS1 regulate CRC progression, metastasis, drug resistance, and DNA methylation. Gut microbiota metabolite, SCFAs, influence CRC development. Targeting lncRNAs and microbiota metabolites holds promise for CRC diagnosis and treatment, including second-generation multi-target stool RNA and DNA tests [44]**.**

Faecal immunochemical test (FIT) remains the basis of colorectal cancer (CRC) screening with high sensitivity (97%) but lower specificity (64.9%) [45, 46]. To enhance detection, multitarget stool DNA (mt-sDNA) tests like Cologuard were developed that incorporate FIT and biomarkers like methylated DRG4 and BMP3 showing up to 90% sensitivity for CRC and 42% for advanced precancerous lesions [47, 48]**.**

Circulating tumour cells (CTCs) and cell-free DNA (cfDNA), especially circulating tumour DNA (ctDNA), show promise for early cancer detection. CTCs exhibit high sensitivity with an accuracy of up to 95.2%, while cfDNA detects mutations and monitors cancer recurrence [49]**.**

Dysregulated snoRNAs, like SNORA51 and SNORD15B, are also potential non-invasive biomarkers for advanced colorectal cancer. SNORA51 was upregulated in CRC patients and, combined with fecal hemoglobin, identified CRC (AUC = 0.86) and advanced adenomas (AUC = 0.68) in FIT + individuals, highlighting the potential for further research [50]**.**

SCAT1, SCAT2, and SCAT8 were significantly upregulated in CRC tissues compared to adjacent non-cancerous tissues. Spearman correlation analysis showed positive correlations between SCAT1 and SCAT8, and SCAT2 and SCAT8, suggesting potential co-regulation or involvement in similar oncogenic pathways. These findings highlight SCAT1, SCAT2, and SCAT8 as potential biomarkers for CRC detection and their role in CRC oncogenesis [51]**.**

CMSmin reduced CRC incidence by 40% and mortality by 52% but less effectively than multi-target stool DNA, annual FIT, or colonoscopy. Its outcomes could match FIT with a participation rate 1.4–1.8 times higher [52]

Blood-based biomarkers for CRC screening must surpass CMS coverage decision accuracy to match the effectiveness and cost-effectiveness of annual FIT or decennial colonoscopy [53]**.**

The USPSTF recommends several colorectal cancer screening strategies for average-risk individuals, including annual guaiac fecal occult blood tests (gFOBT), annual FIT, stool DNA-FIT (Cologuard) every 1–3 years, and colonoscopy every 10 years. Other FDA-approved tests, like colon capsule and methylated serum septin 9 tests, are not USPSTF-endorsed [54]**.**

### Computed tomography colonography (CTC)

#### Technological advances: recent improvements in CT imaging techniques and software for enhanced visualization of colonic lesions

Recent technological improvements in CT imaging have significantly enhanced the visualization of colonic lesions, particularly in CRC diagnosis. Virtual colonoscopy, or CT colonography (CTC), has emerged as a non-invasive alternative to traditional colonoscopy. Advances like iterative reconstruction and deep learning algorithms have improved image quality and reduced radiation exposure, enabling better detection of polyps. Innovations like spectral CT and dual-energy CT have also enabled more accurate differentiation between benign and malignant lesions, while perfusion CT offers insights into tumour vascularization for better treatment planning [55, 56]. 

#### Screening guidelines: comparison of CTC with traditional colonoscopy and its role in CRC screening guidelines

CTC has been compared to traditional colonoscopy in detecting polyps and adenomas. Sensitivity for polyps larger than 10 mm is around 84% but drops to 70% for smaller lesions. Traditional colonoscopy offers near-perfect sensitivity and the ability to remove polyps in real time, which CTC cannot. However, CTC’s non-invasive nature and lower complication rates make it a useful alternative, particularly for those unable or unwilling to undergo traditional colonoscopy [4, 57]. 

### Advantages and limitations

CTC offers several advantages, such as being non-invasive and avoiding sedation, making it quicker and more convenient for patients. However, bowel preparation is still required, and radiation exposure along with extracolonic findings can lead to further interventions. CTC is effective for detecting larger polyps but struggles with flat lesions, like sessile serrated adenomas, which are harder to detect but are significant risk factors for CRC [4].

#### CRC screening guidelines

CTC is considered an alternative for individuals unable or unwilling to undergo traditional colonoscopy. Various CRC screening guidelines recommend CTC at 5-year intervals for average-risk individuals starting at age 50 (or 45, depending on the guideline). For example, the American College of Gastroenterology recommends CTC every 5 years, though as a conditional option due to its lower sensitivity for smaller or flat lesions and the need for follow-up colonoscopy [57]. European and other international guidelines vary but often suggest CTC as a secondary option after stool-based tests like the faecal immunochemical test (FIT) [4].

### Artificial intelligence (AI) in imaging

#### AI algorithms: overview of AI-based tools for polyp detection and classification

AI-based tools have shown great potential in improving the detection of polyps, particularly small and subtle lesions often missed in conventional colonoscopy. Convolutional neural networks (CNNs) play a key role in this by enhancing real-time image recognition. AI systems, such as computer-aided detection (CADe), assist endoscopists by processing image features like texture and shape to detect polyps with high accuracy [58]. AI has been shown to significantly increase both the polyp detection rate (PDR) and adenoma detection rate (ADR), key metrics in CRC prevention. AI improved PDR by 1.75 times and ADR by 1.53 times compared to standard colonoscopy, highlighting its clinical importance in early cancer detection [59].

#### AI in polyp classification

AI has improved not only polyp detection but also classification. Deep learning models help distinguish between non-neoplastic (hyperplastic) and neoplastic (adenomatous) polyps, aiding real-time decision-making during colonoscopy. This AI-based classification is pivotal for decisions regarding polypectomy and histopathological examination [59].

#### Impact on screening efficiency: evaluation of AI’s impact on reducing false positives and false negatives and improving overall screening accuracy

AI has significantly reduced false negatives in colonoscopy by enhancing the detection of small, flat, or subtle lesions, thus reducing the risk of post-colonoscopy CRC (PCCRC). Studies have demonstrated a 50% reduction in adenoma miss rates with AI, decreasing the false negative rate from 29.6 to 6.8%. AI also improves screening accuracy without increasing false positives or adverse events, ensuring greater safety for patients [60].

### Preventive strategies

#### Lifestyle and dietary interventions

Colorectal carcinoma (CRC) is driven by a number of factors which reflect its multi-factorial nature. A sedentary lifestyle, obesity, alcohol and tobacco consumption, chronic sleep deprivation, and a number of other lifestyle-related factors are the common modulators in the risk of CRC [61]. When it comes to food, the most beneficial diet for preventing the colon cancer focuses on high fibre and low-fat intake. The fibre-packed meals contribute to the dilution of colonic contents and optimization of bacterial fermentation which results in an increased production of short-chain fatty acids [62]. In an animal model of CRC, these fatty acids have been noted for their ability to alter a number of cell cycles, cell proliferation, and apoptosis regulators [63]. Foods like vegetables, fruits, grains, and nuts are recognized for promoting gut health and preventing any inflammatory responses. Their protective role also stems from the anticancer phytochemicals that disrupt the cancer cell signalling mechanisms [64]. The B vitamins have also been brought to the spotlight due to their key role in DNA synthesis, repair, and demethylation [65]. Lack of physical activity also contributes to the pathogenesis of CRC. In obese people, the adipose tissue acts as a metabolically active organ that releases cytokines and stimulates T cells that trigger chronic inflammation and insulin resistance [66], which along with IGF-1 (insulin growth factor-1) promotes carcinogenesis [67]. A meta-analysis by Wolin et al. reveals that physically active individuals have around 24% lower risk of CRC development compared to those with sedentary lifestyles [68]. Sleep deprivation is another factor being linked to CRC. Hrushesky et al. have shown that night shift workers, both men and women, have a 50% increased risk of developing CRC [69].

#### Chemoprevention

Recently, there has been an increasing interest in chemoprevention against CRC. The anti-inflammatory agents have shown a chemoprotective effect against colorectal carcinogenesis [70]. Aspirin, a commonly used NSAID, brings favourable outcomes in prevention of CRC by inhibiting cyclo-oxygenase enzymes, COX-1 and COX-2, and bringing down the levels of Prostaglandin PGE2 which is known to promote tumourigenesis [71, 72]. The use of non-aspirin NSAIDs has been associated with an increased risk of GI bleeding, most likely due to COX-1 inhibition and that is why selective COX-2 inhibitors are considered a preferred choice [73]. Since ulcerative colitis lesions are considered to be one of the risk factors in the development of CRC [74], aspirin derivatives such as 5 amino-salicylates have proven to show better results in preventing CRC in such patients [75]. Ursodeoxycholic acid is another agent used that works by inhibiting ACF (Aberrant Crypt Foci) growth and prevents CRC development [76]. Several, commonly used vitamins and minerals are also being studied for their role in CRC prevention.

#### Vaccination

Amongst all these various environmental factors, one such is HPV (Human Papilloma Virus). Although there is much evidence regarding the role of HPV infection in causing anal cancer, not much evidence is available for its association with CRC [77]. A retrospective, controlled study involving the use of dissected colorectal tissues, and adjacent non-tumour tissues from CRC patients as well as from patients without cancer found that HPV DNA-positive cells were present in almost all the CRC lesions [78]. The mechanism through which HPV precipitates CRC is yet to be investigated. However, from the evidence available about its link with CRC, it is imperative to include HPV vaccination in the preventive course of CRC.

## Guidelines and recommendations

### Current screening guidelines: (Table 2)

**Table 2:** Comparison of different CRC screening methods

Screening method	Sensitivity	Specificity	Frequency	Invasiveness	Cost	Advantages	Limitations
Faecal immunochemical test (FIT)	High for late-stage CRC (79–85%)	Moderate (95%)	Annually	Non-invasive	Low	Simple non-invasive	Less effective for early-stage or adenomas
Colonoscopy	Very high (95%)	Very high (99%)	Every 10 years	Invasive	High	Gold standard, polyp removal possible	Requires sedation, bowel preparation
CT colonography	High (90% for polyps > 10mm)	High (85%)	Every 5 years	Non-invasive	Moderate	No sedation, fast recovery	Requires follow-up colonoscopy for positive results
Liquid biopsy (ctDNA)	Moderate (40–80% depending on stage)	High	Varies	Non-invasive	High	Detects early-stage recurrence, non-invasive	Expensive, complex processing
Flexible sigmoidoscopy	High (for distal colon)	High	Every 5 years	Minimally invasive	Low	Targets distal CRC	Does not cover the entire colon

Colorectal cancer (CRC) screening is essential for early detection and prevention, thus.

prominent health organizations have developed evidence-based guidelines to enhance.

treatment outcomes and reduce mortality. The US Preventive Services Task Force (USPSTF),

American Cancer Society (ACS), and American College of Gastroenterology (ACG).

recommend that adults aged 45–75 should undergo routine CRC screening, with selective.

screening recommended for those aged 76–85 [58, 79, 80].

These organization advocate for average risk adults to participate in regular screening.

protocols, which include the following options:

- **Stool-based tests**
- Faecal immunochemical test (FIT) every year
- Highly sensitive guaiac-based fecal occult blood test (gFOBT) every year
- Multi-target stool DNA test every 3 years
- **Structural examinations**
- Colonoscopy every 10 years
- CT colonography every 5 years
- Flexible Sigmoidoscopy every 5 years [80]

ACG also suggests colon capsule every 5 years and septin9 (blood-based test) along with the suggestions above [58]. Furthermore, the ACS recommends a risk-stratified screening approach for individuals with an increased risk of CRC, who may need to start CRC screening before age 45 and be screened more often. These include the following:

- A strong family history or personal history of CRC or certain types of polyps
- A personal history of inflammatory bowel disease
- A known family history of a hereditary colorectal cancer syndrome, such as FAP or Lynch syndrome
- A personal history of radiation to the abdomen or pelvic area to treat prior cancer [80]

### Emerging guidelines

The recent guidelines have lowered the recommended screening age for CRC from 50 to 45. This change responds to a 15% increase in cases among 40–49-year-old between 2000 and 2016 [81]. Notably, CRC screening guidelines vary globally, reflecting differences in healthcare systems, resources, and population needs. The European Society of Medical Oncology (ESMO) recommends screening adults 50–75 with colonoscopy (every 10 years), sigmoidoscopy (every 5–10 years) plus annual FOBT or FIT [82]. In the Asia-Pacific region, FIT and colonoscopy are the main methods for population-based screening, with the starting age at 50 [83], except for Japan which starts screening at 40 [82]. In the South American region, Argentina and Brazil have guidelines which recommend FIT every 2 years for adults ages 50–75 years old [84, 85]. However, screening rates remain low, and guidelines are limited in low- and middle-income countries. 

## Challenges and future directions

### Barriers to implementation

Various barriers to colorectal cancer screening include cost, young age, ethnic and racial minority, recent immigration to the USA, and residence in a rural area [86]. According to NHIS data, a lower rate of CRC screening was found in Hispanics, Asians, the lower socioeconomic class population, recent immigrants, and the uninsured compared to US-born individuals and private insurance holders [87]. The most common causes of barriers to CRC screening in rural areas of the USA were identified to be high screening costs, discomfort in undergoing screening, lack of knowledge of the need for CRC screening, and lack of physician recommendation [88]. It has been found that some patients after they receive positive FOBT refuse to obtain follow-up invasive colonoscopy due to stress and anxiety [89]. Disparities in CRC screening in different populations exist. Higher odds of any colorectal cancer screening were found in Spanish-preferring Latinos compared to non-Hispanic whites [90]. A higher incidence of CRC-related mortality and low CRC screening was seen among African Americans, which was possibly due to higher evidence of medical distrust among African Americans [91]. Disparity in CRC incidence and outcome might occur due to differences in exposure to risk factors, limited access to screening, chemoprevention, and follow-up on abnormal test results. These factors operate at the individual, provider, health system, community, and policy levels to perpetuate CRC disparities [92].

## Future research and innovation

Oral microbiota can be a novel biomarker in the early diagnosis of CRC. If its application can be confirmed, it might progress in the screening program and impact clinical practice [93]. Biomarkers such as circulating tumour cells (CTC) and cell-free DNA (cfDNA), ncRNA/lncRNA are key biomarkers that are used in diagnosis [94]. A blood-based CRC screening test that detects cell-free DNA and circulating tumour DNA has opened up the possibility for a better non-invasive screening approach [95]. Previously stratifying the population for screening based on age and polygenic risk, or in combination with other factors including family history has been found to be efficacious [96]. MISCAN-Colon is a well-established microsimulation model for CRC [97]. It estimates the costs, benefits, and harms of different uniform screening strategies as well as personalized screening strategies which were based on polygenic risk and family history of CRC [97]. The polygenic risk score (PGS) can be generated by incorporating age, sex, and FIT value. It can improve the current CRC screening, most likely for higher at-risk subgroups [98].

## Conclusion

With a variety of invasive and non-invasive techniques available to identify cancer early. To enhance prognosis and lower mortality, colorectal cancer screening has undergone tremendous advancement. Although colonoscopy and faecal immunochemical assays (FIT) are still good standards for detecting colorectal cancer (CRC), advances in liquid biopsy, proteomics, and imaging have transformed the field and offered less invasive, more precise choices, for early identification and surveillance, circulating tumour DNA (ctDNA) and other biomarkers show tremendous potential. Nevertheless, difficulties with accessibility screening, especially in low-resources environments, emphasize the necessity of applying these developments more widely. In the future, lowering disparities, expanding screening availability globally, and customizing preventative measures using risk-based methods will be crucial to lowering the cost of CRC worldwide.

## Data Availability

No datasets were generated or analysed during the current study.
